# Governing Political Realities in NCD Agenda Setting in LMICs: A Case of the Carrot and the Stick? Comment on "National Public Health Surveillance of Corporations in Key Unhealthy Commodity Industries: A Scoping Review and Framework Synthesis"

**DOI:** 10.34172/ijhpm.8836

**Published:** 2024-12-14

**Authors:** Peter A. Delobelle

**Affiliations:** ^1^Department of Medicine, Faculty of Health Sciences, University of Cape Town, Cape Town, South Africa.; ^2^Department of Public Health, Faculty of Medicine and Pharmacy, Vrije Universiteit Brussel, Brussels, Belgium.

**Keywords:** Public Health Surveillance, Unhealthy Commodities Industries, Low- and Middle-Income Countries, Non-communicable Diseases

## Abstract

In their scoping review Bennett et al present a summary framework for public health surveillance of unhealthy commodity industries (UCIs) that impact human health, which is important in view of the rising burden of non-communicable diseases (NCDs), especially in low- and middle-income countries (LMICs). The authors focus on the tobacco, alcohol and food and beverage industry and discuss who should "own" the process; where in the public sector administration the responsibility should lie; and how and which practices or organizations to monitor. They also argue that the monitoring should transition from academia and civil society to (sub)-national governments because of their central role in the protection of public health. This commentary argues that the challenges related to NCD policy-making in LMICs should be viewed from within a political economy perspective and that support for UCI monitoring has to be bolstered by independent accountability mechanisms and rights-based advocacy at national and global level.

 While it is undeniable that governments play a key role as duty bearers for public health monitoring and regulation, in low- and middle-income countries (LMICs) they often face constraints in terms of resources, structure and capacity. As the authors indicate, this may represent a significant obstacle to implementing national public health surveillance of unhealthy commodity industries (UCIs). Apart from an already weaker regulatory environment with more opportunities for industry interference, the question can thus be raised if there is sufficient *capacity* to plan and conduct UCI surveillance at the national level. Government budgets are allocated in view of highly competing healthcare demands, and non-communicable disease (NCD) policies traditionally focus on a downstream prevention agenda. As the slow and uneven implementation of World Health Organization (WHO) “best buys” in LMICs has shown, this depends not only on the ability to define, shape and pass policy into law but also the ability to implement, enforce and monitor these policies.^1^ Monitoring UCIs when resources to enforce NCD legislation are lacking hence risks becoming a tokenistic exercise rather than a potential game-changer.

 Second, policy-making in LMICs is shaped by unique economic and political contexts, and attention needs to be paid to the barriers and enablers of policy regulation. Industry tactics such as institutional infiltration, threat of litigation, and information and coalition management are commonly used and serve as a major barrier to implementing tobacco, alcohol and food-related policies. However, as illustrated by Gomez^2^ in his comprehensive case study analysis of the impact of junk food industries on public policy in emerging economies, industry is only partly to blame for the often quoted lack of regulation. Governments are equally at fault when ambitious presidents in LMICs benefit from colluding with industry to pursue their alternative political, economic and social welfare agendas. The question therefore becomes, is there enough political *will *to go against the interests of powerful UCIs?

 For obesity and diet-related NCD policy, according to Mazzocchi et al,^3^ from an economic point of view, there are only two normative rationales for government intervention: (*i*) the need to address consumption-generated externalities, which in more developed economies are typically covered by public insurance and passed on to consumers, but which can be addressed by taxes; and (*ii*) the ethical imperative to address consumer ignorance (ie, providing health information). In South Africa, ex-ante modeling of the impact of the cost of obesity on national healthcare expenditure informed the adoption of a sugar-sweetened beverages tax in 2018, albeit at a less than optimal level due to industry opposition linked to its alleged and as yet unproven impact on employment and economic growth. Similar tactics are used to delay the approval of draft tobacco and alcohol policy, despite evidence of the impact of tobacco and alcohol on public health and the opportunity for cost savings in the national health budget.

 In South Africa, evidence of tobacco industry interference in policy formulation was also abundant in the era of “state capture” during which government was actively implicated in political corruption and fraud. Van Loggerenberg,^4^ in his tell-all book *Tobacco Wars*, describes how his unit at the South Africa Revenue Service became a “victim of war of powerful industry players and high profile political stakeholders compromised by state capture.” The plot describes spy networks, tax evasion and corruption, and rivalry between political factions and their stakeholders, which include smaller locally owned companies and transnational corporations with shareholders from different social and racial groups blaming each other for the increasing illicit trade in tobacco products. The latter was especially apparent during COVID-19, when a temporary tobacco sales ban affected the illicit trade, but also serves to highlight the dynamic interplay between economic and public health priorities operating in a complex political landscape in a state of flux.

 Given their specific political and economic context, the complexity of NCD policy-making in LMICs can hence not be underestimated and needs to be examined using a political economy lens. Even in a “steady-state” context, corporate political activity (eg, campaign donations) enables favorable decision-making and agenda-setting as reciprocity may be expected once the party is in office.^5^ Collins et al^6^ suggested that active cooperation with the private sector is needed to bring about policy change, to pool resources, and to generate solutions, but the question remains as to how best to “interact, engage or partner with” industry. Buse et al^7^ discussed some of the challenges inherent in public-private engagement, which critics warn will only benefit industry in the absence of clear mechanisms of engagement. New guidance to help this process has been published by WHO, suggesting that public-private partnerships could be successful if safeguards are in place to define the rules of engagement, align incentives to shared objectives, and manage the potential conflicts of interest, acknowledging the diversity of commercial actors as well as their practices and attributes.^8^

 However, rights-based advocacy is needed to support the process, as shown by the South Africa NCD Alliance in their pursuit of more public participation in national NCD agenda setting. Rights-based arguments are important to guide evidence-based interventions, such as the protection of children’s rights from private sector violations for example in alcohol marketing. At a global level, the International Covenant on Civil and Political Rights and the Convention on the Rights of the Child can be used to pressure governments to adopt child-directed marketing restrictions in line with WHO recommendations. UN agencies use legal instruments that have a more or less binding character, which can be used to guide policy-making. Policy levers at the national level include health impact assessments that can be made mandatory as part of socio-economic impact assessment systems, which in South Africa replaced the more generic regulatory impact assessment. Research, however, has shown that industry also interferes with these assessments.

 If there is an area where public health and industry interests intersect, then it could be in corporate reputation, which scores high on the corporate affairs agenda. The food industry, for example, gains substantial reputational benefits from corporate social responsibility (CSR), which is seen as necessary to create value among public stakeholders. Public health actors could leverage these drivers to encourage shifts toward the promotion and production of less harmful products. Attention could be given to the emerging environmental, social, and governance frameworks for responsible investment and to the *social sustainability* of business practice, which is often overlooked but contributes to the objectives of circular economy in line with Sustainable Development Goal (SDG) guidelines for sustainability. Self-reported CSR and sustainability efforts of UCI, however, reveal widespread and strategic use of misleading tactics that require urgent regulatory attention. The tobacco industry, for example, emphasizes its commitment to a global harm reduction agenda by phasing out traditional tobacco products and replacing them with less harmful alternatives, but it continues to aggressively promote tobacco in LMICs. Similarly, social sustainability obligations are often overlooked by the alcohol industry, and CSR initiatives do not contribute to a reduction in harmful drinking.^9^

 Biased attitudes toward commercial sector engagement in health policy are also found in high-income countries, with a clear consensus in regard to tobacco, but less explicitly alcohol, and more mixed responses to the food industry.^10^ Nutrition-related public-private partnerships face opposition and are difficult to form, and evidence of their effectiveness is limited.^11^ Factors that enable or constrain accounting for health in trade agendas also point to potentially significant obstacles in NCD policy-making. Hence, while the central role of national governments in regulating UCIs’ influence should be acknowledged, accountability mechanisms supported by academic and civil society platforms are strongly needed.^12^ Industry may also interfere in this process, as shown in Mexico and Brazil, where the junk food industry hampers opposition through establishing allies within academia and society. By strategically partnering with the government in creating NCD programs, they also garner social support, which further undermines the potential for advocacy coalitions.

 As regards the issue of *where* in public sector administration UCI monitoring should be located, the authors suggest that this could be led by national health departments, with opportunities for an intersectoral approach for prevention and control. Tools and examples exist but require a high level of commitment and attention to context, content, stakeholders, and strategies. Research in Sub-Saharan Africa has shown that multisectoral action in NCD policy development is possible but requires strong coordination mechanisms with clear guidelines for engagement. This could ideally be monitored by an independent entity, such as a health promotion foundation, which in South Africa has been discussed and envisaged as a National Health Commission in the planned rollout of the National Health Insurance to coordinate sectors in implementing a “Health-in-All policies” approach. Different governance and funding models (eg, health promotion levy) have also been discussed, but so far not been realized.

 Finally, as regards to *which *industries to monitor, it is clear that high market concentration of specific UCIs suggests increased structural power relative to national governments and hence warrants a selection of those with the highest share in monitoring, as the authors stated. In South Africa, the food retail sector is highly concentrated; five large chains (domestic and international) dominate the market, with some independent companies challenging certain areas. Analysis of the formal food and beverage industry includes 13 food manufacturers, two non-alcoholic beverage manufacturers, four supermarket chains, and 10 quick-service restaurant chains that are currently assessed using an updated version of the BIA-Obesity tool developed by INFORMAS in an effort to assess and benchmark voluntary policies and commitments of the formal food industry, to address the double burden of malnutrition in a research partnership project (FoodSAMSA).

 Hence, and while acknowledging that national governments play a crucial role in regulating the environment in which UCIs operate, they are facing both opportunities and obstacles. NCD policy development requires high-level political commitment and intersectoral collaboration due to the breadth and fragmentation of ministries involved. Lack of policy coherence has been reported in the agenda on food, tobacco, and alcohol control, which can only be addressed by adopting an intersectoral or “whole-of-government” approach. Shifts are needed in approaches to economic policy, which is unsurprisingly often unreceptive to public health imperatives for UCI regulation, and a robust architecture and support process for holding actors to account for NCD prevention are necessary. Accountability mechanisms exist at the global (and to some extent national) level for tobacco control through, for example, the Framework Convention on Tobacco Control, but there is less appetite for monitoring the impact of alcohol and food industries.

 International trade and investment agreements act as barriers to food environment regulation for public health nutrition, and the integration of more health safeguards in international investment agreements could be useful. Tension also exists at the intersection of tobacco control and trade policy, but due to imbalance in power and influence, and competing frames in global positioning, LMICs are often at a disadvantage, requiring more global approaches to health governance. Calls have been made for a Framework Convention for Alcohol Control and a Framework Convention for Food Systems, which could help strengthen accountability and reduce power asymmetries, taking normative trends toward sustainable development and the increasing push toward “multistakeholderism” into account.^13^ As Beaglehole et al^14^ pointed out a decade ago, this approach could be informed by lessons learned from the HIV/AIDS response and built around pathways for global and national accountability.

 After COVID-19, however, the focus has shifted to the pandemic prevention preparedness agenda in view of global health security, which risks diverting attention from NCD prevention. Efforts are made to include NCDs in the run-up to the Fourth High-level Meeting on the Prevention and Control of NCDs in 2025, but the focus remains on health systems and how to strengthen the integration of NCDs as part of emergency preparedness and response. Voices typically remain silent on the proverbial elephant in the room: the undeniable role of UCIs in fueling industrial NCD epidemics. New models of governance are therefore needed, built around whole-of-government approaches for intersectoral NCD policy, championed by high-level political support and a focus on sustainable development and economies of well-being, using regenerative business models and transparent policies.^15^ If we are to achieve SDG 3.4, however, this approach needs to be underpinned by independent accountability mechanisms, driven by human rights and unambiguous guidelines for rules of engagement with the private sector.

## Ethical issues

 Not applicable.

## Conflicts of interest

 Author declares that he has no conflicts of interest.
